# Specialization training in Malawi: a qualitative study on the perspectives of medical students graduating from the University of Malawi College of Medicine

**DOI:** 10.1186/1472-6920-14-2

**Published:** 2014-01-06

**Authors:** Adam P Sawatsky, Natasha Parekh, Adamson S Muula, Thuy Bui

**Affiliations:** 1 Division of General Internal Medicine, Mayo Clinic, 200 First St. SW, Rochester, MN 55905, USA; 2 Division of General Internal Medicine, University of Pittsburgh School of Medicine, UPMC Montefiore Hospital, Suite W933, 200 Lothrop Street, Pittsburgh, PA 15213, USA; 3 Department of Community Health, College of Medicine, University of Malawi, P/B 360 Chichiri, Blantyre 3, Malawi

**Keywords:** Medical education, Postgraduate medical education, Specialization training, Medical migration, Sub-Saharan Africa, Qualitative research

## Abstract

**Background:**

There is a critical shortage of healthcare workers in sub-Saharan Africa, and Malawi has one of the lowest physician densities in the region. One of the reasons for this shortage is inadequate retention of medical school graduates, partly due to the desire for specialization training. The University of Malawi College of Medicine has developed specialty training programs, but medical school graduates continue to report a desire to leave the country for specialization training. To understand this desire, we studied medical students’ perspectives on specialization training in Malawi.

**Methods:**

We conducted semi-structured interviews of medical students in the final year of their degree program. We developed an interview guide through an iterative process, and recorded and transcribed all interviews for analysis. Two independent coders coded the manuscripts and assessed inter-coder reliability, and the authors used an “editing approach” to qualitative analysis to identify and categorize themes relating to the research aim. The University of Pittsburgh Institutional Review Board and the University of Malawi College of Medicine Research and Ethics Committee approved this study and authors obtained written informed consent from all participants.

**Results:**

We interviewed 21 medical students. All students reported a desire for specialization training, with 12 (57%) students interested in specialties not currently offered in Malawi. Students discussed reasons for pursuing specialization training, impressions of specialization training in Malawi, reasons for staying or leaving Malawi to pursue specialization training and recommendations to improve training.

**Conclusions:**

Graduating medical students in Malawi have mixed views of specialization training in their own country and still desire to leave Malawi to pursue further training. Training institutions in sub-Saharan Africa need to understand the needs of the country’s healthcare workforce and the needs of their graduating medical students to be able to match opportunities and retain graduating students.

## Background

The critical shortage of healthcare workers in sub-Saharan Africa (SSA) has been well-documented [1]. Within Africa, Malawi reports one of the lowest physician density, with only 0.02 doctors for every 1000 people, compared to South Africa (0.76 doctors per 1000 people), the United States (2.42 doctors per 1000 people) and the United Kingdom (2.765 doctors per 1000 people) [2].

One reason for the shortage has been inadequate training of healthcare workers in SSA. To increase physician training, 58 medical schools have opened in SSA since 1990, and the total estimated graduation from all medical schools has increased from 7,861 graduates in 2008 to an estimated 10,000-11,000 medical students each year in 2011 [3]. A second reason for this is shortage is medical migration. In 2011 there were 10,377 physicians practicing in the US who were born in a country in SSA, with migration trends on the rise [4]. In 2007, 40% of the 254 graduates of the University of Malawi College of Medicine were working outside Malawi, half of those were in the UK [5]. Migration of healthcare workers has contributed to the shortage of healthcare workers in SSA.

There multiple and complex reasons for migration. One reason for this migration is that as medical school output increases, retaining physicians leaving for postgraduate training has become a problem [6]. In a 2011 investigation of career intentions among 984 medical students from 6 sub-Saharan African countries, 91% desired postgraduate training and 40% planned to leave Africa for more available and higher quality training opportunities [7]. Medical students at the University of Malawi College of Medicine report similar desires for postgraduate training [8], with the hope for higher salaries, career advancement and job opportunities [9,10].

While motivating doctors from developing countries to work in their country of origin is a complex issue, continuing education and career development continue to be identified as motivations for healthcare worker retention [11]. This is included in suggestions for high-income countries to improve efforts at regulation, diversification of provider skill mix and investment in health professions education [12]. It has been demonstrated that the presence of postgraduate training opportunities improves physician retention, driving recommendations for the establishment of new postgraduate training programs [3]. While the College of Medicine has developed several postgraduate training programs, many students still report intentions to leave the country [8,9]. The purpose of this study was to explore the perspectives of graduating medical students regarding specialization training in Malawi to better understand the factors that influence students’ reported intentions and to impact future efforts to develop postgraduate training.

## Methods

### Setting and participants

In 1991, the government of Malawi established a medical school at the Queen Elizabeth Central Hospital in Blantyre. It was a 5-year program based on community health that led to a Bachelor Medicine and Bachelor of Surgery (MBBS) degree [13]. After completion of their medical training, graduates participate in an 18 month internship at one of the central hospitals in Blantyre or Lilongwe. In 2004, the College of Medicine started 4-year postgraduate programs that led to a Master of Medicine degree in Internal Medicine, Pediatrics and Child Health, General Surgery, Anesthesia and Ophthalmology [5]. The College has since added a degree in Orthopedics, and some programs (Internal Medicine, Pediatrics and Child Health, and Anesthesia) spend 2 years of training at Queen Elizabeth Central Hospital and 2 years of training in South Africa [14]. Although approved in 2004, Obstetrics and Gynecology was not been offered locally. The first intake to have into this program is planned to start training in September 2013.

Currently, all medical doctors graduating in Malawi are employed by the government for at least 18 months while they complete their internship. Afterwards, there are several options for jobs, including working in the public sector for a government-run central or rural district hospital or working in the private sector for a non-governmental organization or other hospital. The private sector does not normally invest in further postgraduate training, but the government does. This drives many doctors to work in rural district hospitals for a few years before seeking post-graduate training.

We sampled medical students in their final year of study at the University of Malawi College of Medicine to ensure adequate clinical exposure and opportunity for consideration of their future careers. We utilized the student class president to recruit participants. Each participant was given oral and written explanations of the study, and was permitted to ask questions about the study before participating.

### Interviews

Participants underwent a semi-structured, one-on-one interview. The interview questions reflected the specific aims of the study, and were developed through an iterative process between investigators at the University of Pittsburgh School of Medicine and the University of Malawi College of Medicine. All interviews were conducted by a moderator with previous training in qualitative methods who was separate from the training program (AS). The final interview guide has been included in the Appendix.

### Qualitative analysis

The interviews were audio-recorded and transcribed. The transcriptions were de-identified and were uploaded to ATLAS.ti version 6 (Scientific Software Development, Berlin, Germany), a computer software program to support the analysis of qualitative data [http://www.atlasti.com]. We used the “editing approach” to qualitative analysis described by Crabtree and Miller [15]. Two investigator (AS and NP) reviewed the first 7 transcripts and developed a codebook. The two investigators independently applied codes to the final 14 transcripts and adjudicated differences through discussion. We entered the final codes into the coding database, analyzed the codes with subsequent quotations, outlined the main themes and chose exemplary quotations.

Prior to the adjudication process, we compared individual coding of the final 14 transcripts to calculate inter-coder reliability using the Cohen’s κ statistic. The total mean kappa value for the assignment of codes was 0.84, demonstrating “almost perfect” agreement [16].

### Ethical approval

This study was approved by the University of Pittsburgh Institutional Review Board and the University of Malawi College of Medicine Research and Ethics Committee. Written informed consent was obtained from all participants.

## Results

Twenty-one of a total 46 medical students in their final year of training participated in interviews (Table 1). All students reported interest in specialization training. Nine students (43%) were either unsure or interested in pursuing postgraduate training in specialties available in Malawi, while 12 students (57%) were interested in specialties not currently offered in Malawi.

**Table 1:** Participant demographics

**Demographic**	**N (%)**
Gender	
Male	13 (62%)
Year in school	
5th year	21 (100%)
Origin	
Blantyre	12 (57%)
Lilongwe	3 (14%)
Other (inside Malawi)	4 (19%)
Other (outside Malawi)	2 (10%)
Career interest	
Emergency medicine	3 (14%)
Surgical subspecialty	3 (14%)
General surgery	2 (10%)
Neurology	2 (10%)
Obstetrics and Gynecology	2 (10%)
Pediatrics	2 (10%)
Clinical pharmacology	1 (5%)
ENT	1 (5%)
Family medicine	1 (5%)
Psychiatry	1 (5%)
Unsure	3 (14%)

Students discussed a variety of themes, including reasons for specialization, impressions of postgraduate training in Malawi, factors in deciding on location of training and recommendations to improve training in Malawi.

### Reasons for specialization

All participants discussed the desire among themselves and their classmates to specialize: “Yeah, I can say everyone wants to specialize. For better quality of life, everybody wants to specialize.” There were numerous reasons for the desire for specialization, including the “natural progression” of their medical training, the opportunity for different jobs, the opportunity for professional impact and recognition, the intellectual stimulation and the perceived need for more specialist doctors in Malawi (Table 2).

**Table 2:** Reasons for pursuing postgraduate specialization training

**Reason**	**Explanation**	**Quotation**
Natural progression	Students discussed the pursuit of specialization training as the “natural progression” of their medical training and part of the culture and expectations	“Our lecturers, our supervisors, they push us towards specializing and they want us to specialize. Most of them will try to push you toward their field of specialty…they expect us to specialize.”
		“Just finishing your MBBS is not good enough…you need to specialize.”
Job opportunities	Students perceived more job opportunities for those with specialization training, including academic, private practice and research appointments	“You’ll be employed by the college as a lecturer there.”
		“Depending on the type of research I want to do, I would have more knowledge in that particular field.”
		“It allows you to do more private practice…there are more opportunities to see more cases…your income is better.”
Professional impact	Students believed that doctors with specialization training held jobs with greater impact	“I would have to specialize to be effective because most of the decision-making is with the consultants as well as the registrars in the hospital.”
Recognition	Students desired the recognition that comes from finishing specialization training and having special expertise	“If I am just a general doctor I don’t think I will be in a position to achieve what I really want. I would like to get somewhere where people can recognize me as a consultant.”
		“…to be recognized for being the only person in your field, or one of the few.”
Intellectual stimulation	Students desired further intellectual pursuits beyond their MBBS degree	“I want to learn more about something in a certain specialty.”
		“We all have a dream that someday we will be a consultant in a certain field. We will focus ourselves to one field where we can direct all our efforts.”
Need for more specialists	Students identified the need for more specialist doctors in Malawi	“There is a burden of disease which it would be nice if we had somebody in that field to explore.”

There were few perceived disadvantages to specialization. Students discussed the increased responsibility and workload with being a specialist: “Now that you are specialized and more trained, a lot of workload would come to you because they expect you to be the more educated one.” It was felt that specialists were not able to work effectively in district settings and they “tend to stick to the central hospitals.” One student expressed concern that this would limit the rural and primary care workforce for Malawi.

### Impressions of postgraduate training in Malawi

Students discussed the positive and negative impressions of the postgraduate training programs at the College of Medicine.

#### ***Positive impressions***

The students perceived many positive aspects of training in Malawi. They believed the learning environment provided ample “exposure” and “experience.” Because of the health care need, registrars have provided care for many patients: “You have a lot more exposure. You see a lot more patients. You do a lot more things than are done abroad.” Registrars were also exposed to a variety of health conditions, many of which present at late stages: “We do have a lot of opportunities here. The amount of pathologies that we get to see here, I don’t think anybody else in the world gets to see that many.” Students felt that this exposure to large numbers of patients with diverse pathology provided a rich learning environment.

Students perceived that registrars developed adequate experience, especially with practical patient care and procedural skills. This hands-on experience was important: “I would want to learn here rather than go outside. Here there is more handiwork involved, hands on work with the patients.” Students felt “there is so much exposure doing practical things,” and saw their comfort level with procedural skills when compared to trainees from other countries.

Lastly, the students discussed the benefit of the structure of the programs. “You do some years here and then some years [in South Africa]. I would prefer to do this because you get the best of both worlds.” Most students liked the idea of training at the two different sites, because they “are exposed to the medicine that we are practicing here and also some of the technologies in South Africa that might not be seen here.” They felt that the time spent training in South Africa could “make up the difference” for the lack of resources and technology in Malawi.

#### ***Negative impressions***

Students discussed several negative impressions of the learning and work environment of postgraduate training opportunities at the College of Medicine and Queen Elizabeth Central Hospital (Table 3). Contained within each of these categories was the overarching theme of limited resources, the main perceived limitation of training in Malawi. Students were “frustrated” and “depressed” with inadequate technology, investigations, medications, supplies, hospital capacity, and staff that limited the training environment in Malawi. “If you have been trained here, you are limited in terms of investigations. You go up to a point, and then you don’t know what else you can do from there.” They were concerned with working in a setting with advanced equipment: “You are not familiar with the equipment and you might look dumb.” One student discussed feeling inadequate because “maybe in ten years we’ll advance to a position to use those investigations.” Students also believed that some departments had inadequate staff for clinical work and trainee education.

**Table 3:** Negative impressions of postgraduate specialization training in Malawi

**Impression**	**Explanation**	**Quotation**
Learning environment		
Limited variety of cases	Students felt that they are exposed to a limited variety of cases, which limited their ability to form broad differential diagnoses	“If you are not careful, there is this knee jerk reaction where every fever is malaria, every cough is TB, every headache is meningitis…exposure to other disease processes makes a difference.”
Same teachers throughout training—limited knowledge	Students discussed that being exposed to the same teachers throughout their training limited their pool of teachers and available knowledge	“It’s a bit too much for some people. You go through your [medical school training] with the same consultants. Then you do your internship training with those same people. And then you do your master’s program under those same people. So, you’re limited for who you can go to for knowledge. And if it’s someone you don’t get along with, it makes it difficult.”
Same teachers throughout training—bias	Students discussed how having the same teachers throughout their training can perpetuate biases	“The tutors here already have a biased view towards the students that they have seen through the years.”
Halted progression of registrars	Students noticed that some registrars have not progressed through their training and graduated on time	“Some registrars don’t seem to be progressing. I hear some registrars have even given up. I don’t want to go through the same. I want to progress.”
Limited scope of training	Students perceived that the training in Malawi only trains registrars to practice in Malawi, limiting the scope of their training	“These programs are designed to train people for the country they are in, specifically the MMed here. You want to have doctors in Malawi. So the objective may not necessarily be to have the best MMed program in the region…you end up saying, 'Would that be adequate?’ I don’t think it is.”
Limited credentials—unable to work in other countries	Students desired to have the credentials to work anywhere in the world once they are done with their specialization training	“There is a catch to the programs, as if you are limited to work in sub-Saharan Africa. [Fellow students] are not really in favor of that, because I think most people want to work abroad in different countries. I think they feel the programs are holding them back.”
Difficult learning environment	Students perceived the learning environment as unnecessarily difficult	“Most people are reluctant to do their masters here because they are making it really tough.”
Limited number of specialties	Students wanted more opportunities for specialization in Malawi	“Here in Malawi, there are only a few specializations. So they are very limited compared to the different specialties I saw outside of the country.”
Contractual obligations	Students felt the contractual obligation to government scholarships was excessive	“I would like to shy away from the programs that they’re offering because of the contractual clause that you have to be in service to the government.”
Consultant shortages	Students perceived that there were not enough clinicians to handle the clinical and teaching responsibilities	“I don’t think there are enough local consultants in most of our departments for them to say that we have adequate doctors to teach our new doctors.”
Working environment		
Overworked	Students felt that the registrars worked harder than was reasonable	“We see our registrars. They are there for quite long and they are overworked.”
Not appreciated	Students perceived that there was a lack of appreciation for the work that is being done by the registrars	“There are lots of patients to see, and maybe they feel that are not appreciated at the end of the day…Everyone is frustrated at the end of the day because it is just a lot of workload.”
Compensation	Students felt that the amount of money paid to registrars was inadequate to pay for expenses	“When you compare the money that [registrars] earn per annum to the cost of school and everything else, you can’t afford it. So you need a scholarship to do postgraduate training, but to get those scholarships is really quite hard.”

When reflecting on the resource limitations, some students even discussed hopelessness towards change: “I don’t think there is anything we can change, but we just have to accept what we have. Even if we contact the ministry of health, they won’t change anything.” These feelings of frustration, depression and hopelessness, linked to limited resources, were the most cited negative impressions of postgraduate training in Malawi.

### Factors in deciding on location of postgraduate training

There were many reasons that were given for staying or leaving Malawi to pursue postgraduate training.

#### ***Reasons to pursue training in Malawi***

Many of the students acknowledged the need to work in Malawi to help their country. One of the main reasons for staying was the belief that to work effectively in Malawi, it is better to train in Malawi: “If you train here you can better serve these people.” This was because “you are more familiar with what actually happens in Malawi.” Some students desired to research tropical diseases: “I want to train here because I want to work in a tropical setting. There is a lot of research to do in tropical countries, like Malawi.” Students viewed training in Malawi for altruistic reasons: “People stay here because they want to help, not because they are happy with the opportunities here.”

Many students also gave personal reasons for staying, including support from family and friends, cost, and the comfort of remaining in a familiar setting.

#### ***Reasons to pursue training outside of Malawi***

Students expressed the desire to leave Malawi for training, expressing a desire to acquire new experiences, to bring experiences back to improve medical care in Malawi, to train in specialties not offered in Malawi, to earn more money, to obtain a better education, or to leave the country (Table 4).“It’s painful for one to leave his country. It is, especially for Malawian people. But you do it so you can have a better life.” Students see specialization training as the optimal time: “It’s hard to leave Malawi. Maybe the best way is through training.”

**Table 4:** Reasons for pursuing postgraduate specialization training outside of Malawi

**Reason**	**Explanation**	**Quotes**
Acquire new experiences	Students desired to have exposure to new clinical settings and different cultures to gain broader knowledge and experience	“I have never been outside of Malawi. I would like to see other things, how people do things outside.”
		“You get more exposure to what’s out there, the differences, and you get to widen your knowledge.”
		“[Outside Malawi] you don’t just read about it, you get to see it.”
		“…also cultural experiences and meeting new people. It broadens your mind and helps you be a holistic doctor.”
Improve medical care in Malawi	Students believed that training elsewhere and returning would better equip them to advance the medical field in Malawi	“I want to see what else is out there so I can work alongside with that and see what I can bring back from there that will be helpful for Malawi.”
Train in specialty not offered in Malawi	Students expressed the aspiration for further training in specialties not currently offered in Malawi	“The only thing that would not keep me here is the lack of the learning opportunity.”
		“There are a lot fields, like Psychiatry for example, that are not offered under the College of Medicine.”
Earn more money	Students did not think that the salaries of registrars was adequate to support their families	“Financial security becomes a problem. If I was to have an option to earn more than I can earn here as a junior doctor, I will choose that. [Elsewhere] the pay is much better than here.”
		“Patriotism does not pay in Malawi…How can I pay my kids’ school fees? It is not right that my father gave me an education and then in the name of patriotism my children suffer.”
Obtain a better education	Students perceived training in other countries as higher quality that training in Malawi	“I think Malawians should not get second class anything. I want to get decent training and give them the best care. If I am teaching medical students, if I did my postgraduate training [outside Malawi], the way I teach that topic will be different…We need quality specialists.”
		“The most obvious choice for anyone would be to leave Africa and go a place that’s offering a better education.”
Leave the country	Many students viewed training in another country as a means to leave Malawi	“Most people that have grown up here think it hasn’t been much fun growing up in Malawi. It’s been tough, so you want to see something different.”
		“If I was to specialize, let’s say in Australia, then I could work anywhere in the world, not just in Malawi.”

### Recommendations

Students discussed several recommendations to improve postgraduate training in Malawi, including improvements in career counseling and mentorship, and changes to the structure of the programs.

#### ***Career counseling and mentorship***

At this point in their training, many students felt that they did not have a very good understanding of the options available for postgraduate training. Many discussed not knowing who to ask about career options and not feeling comfortable discussing them with faculty members or registrars. They believed they would graduate medical school without a clear understanding of their career path:

“When we start our clinical years we should already be thinking about [postgraduate training]. I wish I had the opportunity to talk to people about such things as internship is only one and a half years. It’s not exactly a long time to start making plans…If there is no one to tell us that these options are available, none of us will think of them. We are just going to continue doing what we’ve seen other people do.”

Students wanted guidance from faculty members and registrars on career planning, specialty options, requirements, and scholarships. Without mentoring, people “waste time” trying to figure out options on their own or through word of mouth. Students felt a structured mentoring program would help students with decisions about career planning and postgraduate training.

#### ***Specialization program changes***

Students desired more support for the postgraduate training programs in Malawi. They recommended several changes, including providing more options for training, increasing the number teaching staff, retaining expatriate teachers, improving support and respect, improving working conditions, standardizing the specialization training across Africa, broadening degree recognition, and consolidating training in Malawi versus expanding time in South Africa (Table 5).

**Table 5:** Recommendations for improving postgraduate specialization training programs in Malawi

**Recommendation**	**Explanation**	**Quotes**
More training options	Students felt more specialty options would improve the opportunities for training in Malawi	“Ideally, if we had a wider scope of fields within Malawi to train, that would be the best way.”
		“I am not very keen on specializing here because the options are limited…Here there are less than ten options of things you can do. So, if I was to make it better, I would increase the options.”
		“I think people would be able to stay here in Malawi and it would be cheaper to specialize in your own country.”
Increase teaching staff	Students recognized the need to increase the number of staff for teaching at the postgraduate level	“The College of medicine should make sure each department is well-staffed…Isn’t it possible in some institutions they have people who are just special for teaching?”
		“I would want to make sure we have adequate teachers and staff for [postgraduate training].”
Retain expatriate teachers	Students felt that the expatriate teachers play a vital role in their training, and that there were not enough academic Malawian-trained physicians to support training	“We have concerns because some of the consultants from other countries have been told that their contracts are not being renewed…They say that we have enough doctors in Malawi and enough teachers, but we feel these are the people that are key in training us.”
		“It seems like there is a move to replace a lot of expatriates with local consultants. I don’t think that makes sense.”
Improve personal support and respect	Students perceived the need for more support from faculty members, including increased respect	“…if people could appreciate [trainees] more and understand they are qualified.”
		“I think if [the consultants] really encouraged the students and were nice, most people would be willing to do more of their further studies here.”
		“Give [trainees] the right environment where they can grow. And also motivate them.”
Improve working conditions and compensation	Students desired improved working conditions, better resources for working and training, and better compensation	“So if something was to change, I think it would be the working conditions. Mostly if the salary was better.”
		“The one thing for me would be the resources. I don’t like to hear about, 'Oh, other countries do this,’ and I can’t even see what that thing looks like. I would rather be there using it and learning how it works.”
Standardize training across Africa	Several students discussed the need for standardization of the training programs across countries within the region	“Even starting, like [College of Surgeons in East, Central and South Africa (COSECSA)], starting the same thing in other departments. As long as the qualification in the end doesn’t require you to specialize further when you go outside the county.”
Broaden degree recognition	Students would like to be credentialed to work anywhere in the world after completion of their training	“I guess the programs need to be changed so that they’re internationally recognized, at least so that you can work in other countries.”
Consolidate training in Malawi	Some students saw the benefit in having all of the training provided in Malawi, instead of spending some of the time in South Africa	“If you are planning to work in Malawi, I think you should train here the whole 4 or 5 years.”
		“When they are doing their second half, the department loses registrars and it’s hard on the people that are left behind.”
Expand training in South Africa	Many students saw the benefit of having a portion of their training in South Africa, and some even desired to spend more time there during training	“I think you should have more time experiencing all these other advanced things rather than getting more of your time here in a place with limited resources.”

Students recognized that medical training in Malawi is evolving and requires constant evaluation: “The College of Medicine has to decide what kind of doctors they want to train. What do we want? What’s the vision after we train them? Do we want them to specialize? If we do, how much can we do?” Students believed that these questions should direct future revisions of postgraduate training in Malawi.

## Discussion

The goal of this study was to characterize medical students’ perspectives of specialization training in Malawi to elucidate factors that influence these reported intentions and to stimulate future efforts to develop postgraduate training. Our investigation confirms findings from previous studies, which revealed that even after the development of postgraduate opportunities at the University of Malawi College of Medicine, many graduating medical students still want to leave Malawi to pursue postgraduate training [8,9]. All of our participants desired to pursue specialization training because of the perceived personal and professional benefits and to align with peer and faculty expectations. They provided positive and negative impressions of the postgraduate training programs, stating the benefits of exposure and experience while outlining many of the problems deriving from working in a setting with limited resources. They believed that many stayed in Malawi for more altruistic reasons, giving many more reasons to leave for further training. Students perceived many areas of improvement in postgraduate training in Malawi, and offered several recommendations for improving the quality of training.

The retention of physicians in Malawi and other sub-Saharan African countries is an important part of building the healthcare workforce; developing adequate postgraduate training opportunities is one important piece of that puzzle [1,3]. Even 6 years after the development of postgraduate programs in Malawi, only 11.8% of graduating medical students intended to specialize in Malawi [17]. Our study helps to explain why despite efforts to build postgraduate training programs in Malawi, many graduating medical students still desire to leave the country. Students suggested drawbacks of the current training programs, limitations in options for specialization training, and desires to leave the country as reasons to leave Malawi for postgraduate training. While improving postgraduate training opportunities may address the limitations of the current system, it may not completely change the perceived need to leave Malawi.

Graduating medical students in Malawi perceived their limited understanding of training options and desired mentoring during medical school. They have presented several contradictions, including the impression that Malawi is the best place to train for those who want to work in Malawi against the stated intention of the majority of students to train outside of Malawi and then eventually return to work in Malawi after training. While students felt that the limited resources in the training program limited their education, they also felt that training with limited resources prepared them to work in that same environment. On a deeper level, this demonstrated a conflict between the interests of the country (seeing the need to train and work in Malawi) and the individual (wanting to improve their situation or education). This was sometimes reconciled by the desire to bring back new experiences and expertise to help improve their country, but conflict remained. The World Health Organization recognizes this conflict in the need for “balancing the freedom of individuals to pursue work where they choose with the need to stem excessive losses from both internal migration (urban concentration and rural neglect) and international movements from poorer to richer countries [1].” Our study revealed the expressed needs of students and many changes that could be made to the system to help stem excessive losses.

Students recognized that the government and the College of Medicine need to “decide what kind of doctors they want to train.” While many students graduating today desired specialization training, specialist care has yet been shown to improve health care in Malawi. For instance, students in this study recognized that there is little support for specialists in rural areas, and therefore many specialist trained doctors will remain in cities. They also recognized that increased specialization training may not address the need for primary care in the country. This study provides optimism that the College of Medicine’s efforts at developing a master’s program in family medicine can fulfill students’ desire for specialty training and fill the gap in rural–urban healthcare inequalities. Further developments in specialization training need to consider the question regarding the numbers of specialists and other healthcare workers that are needed to support healthcare in Malawi.

There are several limitations to our study. First, this study was conducted at a single institution, which may limit transferability to other settings. Second, we only interviewed medical students, and impressions may change over time as they progress through internship. Interviewing graduating medical students allowed for adequate experience and accessed trainees at a pivotal time in their training. Third, there is a difference between student intentions and their eventual actions. Fourth, the primary author (AS) played a large role in conducting interviews, coding transcripts and analyzing data. To help minimize bias, the coding was performed independently by two authors, achieving excellent inter-coder reliability. All analysis was done using a team approach to help minimize bias.

## Conclusions

Students graduating from medical schools in sub-Saharan Africa desire postgraduate specialization training and training programs are being developed to retain physicians within their home countries. Despite those efforts, students graduating from the University of Malawi College of Medicine have mixed views of postgraduate training in their own country and still desire to leave Malawi for specialization training. Universities and hospitals in sub-Saharan Africa need to understand the needs of the country’s healthcare workforce and the needs of their graduating medical students to be able to match opportunities and stem “excessive losses” of trained physicians.

## Appendix

### Interview guide

#### Introduction

1) What motivated you to choose to go to medical school?

2) What are your plans for after you graduate from medical school?

#### Available options

3) What options are available to medical students graduating from the Medical College of Malawi for further training after medical school?

[Prompt] List what options are available.

4) How did you find out about these options?

5) When thinking about [insert potential option], what makes it a desirable or undesirable option?

[Prompt] What do your classmates say about this option?

[Prompt] What do former classmates say about this option?

[Prompt] What do your faculty say about this option?

6) How did/would you choose among those options?

#### Post-graduate training

7) What are your options for post-graduate training after medical school?

8) What factors play a role in your decision regarding pursuing or not pursuing post-graduate training after medical school?

[Prompt] What is/was the most important factor in making your decision?

9) What do you know about the masters programs at the University of Malawi School of Medicine?

[Prompt] What are your impressions of training in Malawi after medical school? [Prompt] What have you heard about training in Malawi after medical school?

[Prompt] What are your impressions of the masters programs?

[Prompt] What do the registrars say about those programs?

[Prompt] What benefits come from doing the master’s program?

[Prompt] What factors influence you or other students you know to pursue or not pursue further training in Malawi?

10) What opportunities are there to pursue post-graduate training in another country in Africa?

[Prompt] Do you know anyone who has done this? What did he/she say about it?

[Prompt] What have you heard about these opportunities?

[Prompt] How available are these opportunities? How do you pursue them?

[Prompt] Do you know people who came back after pursuing this opportunity? What jobs were available when they came back?

11) What opportunities are there to pursue post-graduate training in another country outside of Africa?

[Prompt] Do you know anyone who has done this? What did he/she say about it?

[Prompt] What have you heard about these opportunities?

[Prompt] How available are these opportunities? How do you pursue them?

[Prompt] Do you know people who came back after pursuing this opportunity? What jobs were available when they came back?

12) What would need to change to make pursuing further training in Malawi more enticing for you or your fellow medical students?

#### Beyond training

13) What job opportunities are available in Malawi after training?

14) How does the type of post-graduate training you receive affect the types of jobs available?

15) What do you think about working in rural areas (village/district areas) within Malawi?

16) What do you think about required service to serve in district areas in Malawi? What are the benefits and harms to you as a trainee? What are the benefits or harms to your country?

#### Summary

17) If you were to make one change to the way medical training after medical school was delivered in Malawi, what would you change?

18) Is there anything else you would like to share on this topic?

## Competing interests

The authors declare that they have no competing interests.

## Author’s contributions

AS contributed to the conception and design of the study, carried out all of the interviews, participated in the coding and analysis of the data, and drafted the manuscript. NP participated in the coding and analysis of the data, and assisted in drafting the manuscript. AM contributed to the conception and design of the study and to the analysis of the data. TB contributed to the conception and design of the study and to the analysis of the data. All authors read and approved the final manuscript.

## Pre-publication history

The pre-publication history for this paper can be accessed here:

http://www.biomedcentral.com/1472-6920/14/2/prepub
